# Integrated Systems Biology Pipeline to Compare Co-Expression Networks in Plants and Elucidate Differential Regulators

**DOI:** 10.3390/plants12203618

**Published:** 2023-10-19

**Authors:** Nilesh Kumar, M. Shahid Mukhtar

**Affiliations:** Department of Biology, University of Alabama at Birmingham, Birmingham, AL 35294, USA

**Keywords:** gene expression, biological networks, co-expression, regulation, TF–target

## Abstract

To identify sets of genes that exhibit similar expression characteristics, co-expression networks were constructed from transcriptome datasets that were obtained from plant samples at various stages of growth and development or treated with diverse biotic, abiotic, and other environmental stresses. In addition, co-expression network analysis can provide deeper insights into gene regulation when combined with transcriptomics. The coordination and integration of all these complex networks to deduce gene regulation are major challenges for plant biologists. Python and R have emerged as major tools for managing complex scientific data over the past decade. In this study, we describe a reproducible protocol POTFUL (pant co-expression transcription factor regulators), implemented in Python 3, for integrating co-expression and transcription factor target protein networks to infer gene regulation.

## 1. Introduction

For their growth and development under a wide spectrum of environmental conditions, diverse organisms including plants and animals rely on the regulation of finely tuned gene expression [1]. An indispensable component of gene regulation is transcription factors (TFs), which interact with other regulatory constituents to form gene regulatory networks (GRNs) that govern a variety of cellular processes [2,3,4]. In addition to regulating mRNA levels, GRNs often interact with metabolic networks and environmental cues to specify spatial and temporal patterns [3,4]. However, it remains unclear what exactly drives the correlated expressions between groups of genes [5]. Even under relatively stable physiological conditions, i.e., *Drosophila melanogaster*, the collective profile of gene expressions in each cell type or tissue does not remain static, since genes are continuously regulating each other [6,7]. When multiple TFs target the same genes, co-expression is accentuated, and exhibits time-shifted behavior; this tendency increases if multiple TFs target the same genes [8]. Transcriptional/translational feedback loops control the growth and development of plants [9,10]. Similarly, various aspects of plant biology are influenced by the GRN, including gene expression, metabolism, developmental processes, and responses to stress [11,12,13,14,15,16]. In addition, most TF–target interactions rewire transiently in response to stimuli, while a few acts permanently as hubs. The concept of network centrality and network motif has been used in the past, such as in [17,18,19,20], in order to study GRNs and their rewiring under stimuli.

In addition to GRN inferences, co-expression networks are useful for understanding gene regulation [17,21]. Through co-expression network analyses, genes with similar expression patterns across varying [22] or non-varying [23] conditions can be discovered, clustered, and explored simultaneously. Often, genes that encode proteins from the same pathway or complex co-regulate, even if their functions are unrelated [24,25]. A co-expression network inference consists of three basic steps: (i) estimating the pairwise correlation coefficient from gene expression profiles; (ii) eliminating low-correlation gene pairs; and (iii) clustering the genes into modules or clusters [22,26,27]. An edge and node network are constructed based on genes and gene pairs above a certain threshold [5,28]. Genes participating in the same regulatory pathway or sharing the same function tend to display similar expression profiles, leading to modules or clusters. To provide researchers with system-level resources, several comparative co-expression databases have been developed in the last few years [25,29].

The weighted gene co-expression network analysis (WGCNA) method (also R packages) for the inference of co-expression networks [30] provides the option of constructing the co-expression network by either inferring highly positive edges only or both highly positive and negative edges [31]. Using public datasets of uncut roots and 3-h post-decapitation tissue samples, we investigated underlying genes involved in root regeneration [32]. In our pipeline, co-expression and GRN are determined separately using expression profiles. Then, a network-based integrative approach is used to merge the inferred co-expression and GRN [33,34]. After removing non-overlapping edges, a shared network becomes a co-expressed regulatory network (CERN). As a result, the CERN overcomes the shortcomings of both the co-expression network and GRN, namely the direction of regulation and evidence of co-existence. In addition, CERN-uncut and CERN-(3 h post-decapitation) were compared to discover universal elements involved in root regeneration. It is possible to extend this pipeline to any plant species to integrate gene regulatory networks and co-expression networks, depending on the availability of the TF–target network. Inter-species comparison can be accomplished using a common ID system for homologous genes and proteins.

## 2. Results

In response to multiple stresses within a cell type, the POTFUL pipeline is expected to retrieve a set of genes that are involved in regulating cellular function directly or indirectly. Stress responses in plants are tightly correlated with developmental processes, but their interactions are poorly understood [35,36]. Using an integrative approach, the POTFUL pipeline combines the GRN and co-expression to figure out the omnipresent regulator between samples that are known TF–target pairs and co-expressed together. For the co-expression network to be merged with the GRN, its edges first need to be duplicated so that bi-directional information can be stored in the memory. If nodes A and B have a common edge (e.g., A → B), then an edge (B → A) will also be created. As TF–target edges are compared to edge-duplicated co-expression networks, only the common edges are retained for the further purposes of analysis, while TF–target directionality is preserved for the purposes of further biological interpretation. In the current study, we obtained transcriptome datasets that are derived from two different environmental stresses, i.e., altered uncut and 3 h post-decapitation deprivation conditions. At first, we inferred co-expressions for uncut and 3 h post-decapitation conditions using the expression data of respective samples. For co-expression network inference, the WGCNA R (v1.72-1) package was used, which clusters genes by their expression profiles unsupervised. The minClusterSize parameter in the cutreeDynamic R function can be optimized to adjust number of WGCNA modules or clusters (see Appendix C, Problem 2) [37].

**Figure 1:**
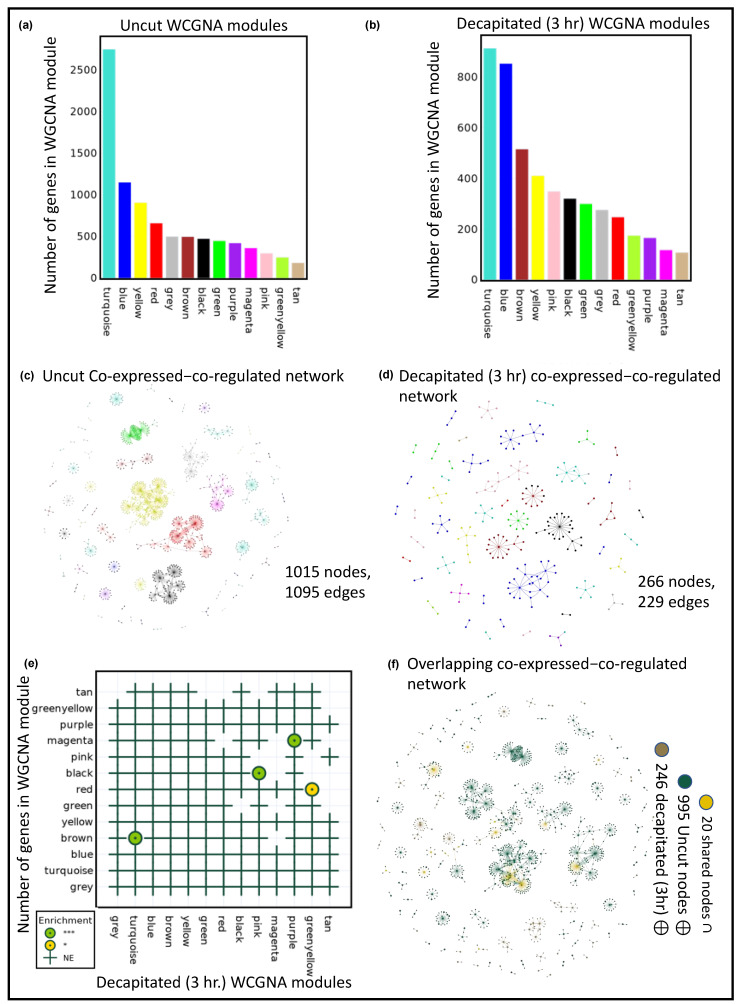
POTFUL analysis of uncut and 3 h post-decapitated samples. A bar plot showing the numbers of genes found in WGCNA modules for uncut (**a**) and 3 h post-decapitation (**b**). The co-expressed–GRNs of uncut (**c**) and 3 h post-decapitation (**d**). (**e**) Module-to-module comparison of gene co-expression networks, enrichment analysis. The dot-plot of enrichment shows three colors of dots/circles: green for *p* < 0.001 (***), and yellow for *p* < 0.05 (*). Where NE stands for not enriched. (**f**) The colors of the nodes in the co-expressed–GRNs overlap indicate exclusive (uncut: green and 3 h post-decapitation: gold) and shared nodes (yellow).

The uncut co-expression network encompasses 8921 nodes and 563,244 edges, and is composed of 13 diverse modules. In the case of a 3-h post-decapitated co-expression network, we obtained the same number of WCGNA modules, e.g., 13, which contain 4756 nodes and 166,625 edges (Figure 1a,b). Generally, more samples lead to more robust and refined results, so 30 uncut samples have a larger co-expression network than the samples taken three hours after decapitation. Thus, the number of overlapping modules would be few or none between two networks of different sizes. Uncut samples are also expected to exhibit more co-correlated gene expression patterns, so the uncut co-expression network contains more nodes than decapitated samples. POTFUL determines which WGCNA modules are overrepresented within one network based on their co-expression in two other networks. A significant correlation exists between the four uncut and 3 h post-decapitated co-expression modules (brown: turquoise (q-value = 3.23 × 10^−7^), red: green-yellow (q-value = 1.726343 × 10^−2^), black: pink (q-value = 1.475838 × 10^−11^), magenta: purple (q-value = 2.583919 × 10^−7^)) (Figure 1e). In the enrichment dot plot, the significance of enrichment is depicted using three colors of dot/circles, *p* < 0.001 (***): green, *p* < 0.01 (**): gold, and *p* < 0.05 (**): yellow. Moreover, the names of modules (by color) are arranged, left to right and bottom to top, starting with “turquoise”, “blue”, “brown”, etc. It ensures that the most important modules are cornered at the bottom left of the enrichment dot plot. Among the combinations, the blue (3 h post-decapitation): yellow (uncut) combination is the most important, since they are high-priority modules and significantly enriched, *p* < 0.001 (Figure 1e). The gene regulatory network (GRN) is inferred from gene expression data using the GRNBoost algorithm (GRNBoost2 from Arboreto (v 0.1.6) Python package) [38,39,40,41,42].

GRNBoost2 produces plenty of putative ranked/weighted (importance score) TF–target integrations. False positive TF–target pairs can be pruned in a variety of ways. Using weight filters (importance) when exporting GRNBoost2 results would be one simple way to filter. To eliminate false positives, we removed the GRN edges that did not appear in the curated TF–target network. If a known TF–target network is not provided, POTFUL will not filter putative GRN edges, or this feature can be turned off by setting Filter to ‘0’ or ‘false’ in the ‘TF_reg’ function.

As a result, uncut (1015 nodes and 1095 edges) and 3 h post-cut (266 nodes and 229 edges) “co-expressed–GRN” networks were constructed. The number of genes in the turquoise module for both the uncut and decapitated (3 h) is the highest., whereas the number of TF–target pairs in the “co-expressed–GRN” network is a maximum in the yellow module (TF:44 and Not TF–target:204) for the uncut network; for decapitated (3 h) network, the maximum TF–target pairs are in the blue module (TF:23 and Not TF–target:48) (Figure 1c,d). There are 20 shared nodes (yellow), 995 unique uncut nodes (green), and 246 decapitated (3 h) unique nodes (gold) in the combined co-expressed–GRN (1261 nodes and 1321 edges) (Figure 1f).

Functional enrichment analysis was performed on an overlapping-co-expression–GRN (Figure 1f and Table S1) using the Metascape tool [43]. Among all 20 shared nodes (yellow) in the overlapping co-expressed–GRN, three genes (AT2G18380/GATA20, AT2G45660/AGL20, and AT3G01530/MYB57) are involved in the reproductive shoot system development biological process. Genes unique to only the uncut (green) samples are significantly enriched (Log(q-value) < −1.3), with “negative regulation of response to salt stress”, “positive regulation of DNA-templated transcription”, “regulation of secondary cell wall biogenesis”, “regulation of defense response”, and “cellular response to hypoxia”. Interestingly, the unique gene (gold) was significantly enriched (3 h post-decapitated) in “positive regulation of cellular biosynthetic process”. The enrichment of post-cut samples in “positive regulation of cellular biosynthetic process” correlates with the original experimental design; that is, after decapitation, it can be expected by the plant to shift its focus more on biosynthesis-damaged tissues to support regeneration.

## 3. Discussion

We investigated the transcriptional data of plants as a case study and created a co-expression network followed by a GRN. The co-expression modules were also compared statistically using WGCNA modules as a basis for the comparison. In addition to the co-expressed pairs, a significant number of these pairs were also found to be included in the GRN. For multicellular organisms such as plants and animals to respond to stress, thousands of genes and their regulatory coordination are required [44]. Often, some genes co-express and act as transcriptional regulators, directly or indirectly. Several purposes can be served by gene co-expression networks, including identifying regulatory genes, prioritizing candidate genes for diseases, and annotating functional genes. Correlation networks cannot identify causality or differentiate regulated genes from regulatory genes. The POTFUL pipeline goes beyond traditional co-expression network analysis by combining differential co-expression analysis with co-expressed–GRNs [45,46]. The applicability of POTFUL is not limited to plant species. Due to their role in phenotypic differences, in different disease states, tissue types, and developmental stages, POTFUL can identify genes with different co-expression partners (see Appendix D). As more omics layers are considered, such as protein–protein interactions and methylome analysis, further enhancements can be made to these genes’ regulatory roles. In summary, POTFUL is a useful tool for comparing pairs of co-expression networks, but it is important to be aware of its limitations before using it (see Appendix B, Limitations).

## 4. Materials and Methods

It is sufficient to have the expression profiles of two differentiating bio-samples (e.g., uncut and decapitated), the TF–target interaction network, and a list of all transcripts relating to the organism being studied. Apart from the computation device, there is no need for reagents or other materials. As part of this protocol, all analysis steps are carried out using Python, an interpreted, high-level general-purpose programming language that can be used on a wide variety of operating systems, including LINUX, Windows, and macOS. Currently, the protocol is written in Python version 3.10.4 on a Linus-based system (Red Hat Enterprise Linux version 7.9 (Maipo)). In addition to Python, R packages and tools are required for several analyses such as WGCNA, though those analyses are not part of this protocol. The protocol can be run on most UNIX and Linux distributions; however, Ubuntu 22 and Fedora 36, Red Hat 7, and macOS Monterey are recommended. In addition to HPC devices, this protocol has been tested on other devices, with the following specifications:

- OS: Windows 11 (5.10.102.1-Microsoft-standard-WSL2), and Fedora 36;
- RAM: 16 GB;
- SDD: 256 GB;
- CPU: Intel i7;
- Conda 4.12.0.

The conda environment file ‘POTFUL.yml’ can be used to install all the necessary Python packages, as explained in the Section 4.

Furthermore, this protocol requires a TF list, TF–target graph/network data, and transcriptomic data. In this protocol, GRNs were inferred using grnboost2 (genie3), and among other possibilities for denoising GRNs, we used a curated TF–target network. The TF–target interactions used in the case study for *Arabidopsis thaliana* were collected from various sources to combine and make a comprehensive TF–target network. This includes an Arabidopsis thaliana regulatory network (AtRegNet) [47], a plant cistrome database (DAP_seq) [48], an Arabidopsis transcriptional regulatory map (ARTM) [48], Curated_1 [49], TF2Network (Curated_2) [50], and Ath [47,49,50,51]. Furthermore, the case study uses RNA-seq data from uncut meristems and root meristem stumps, post-cut (GSE74488). Uncut root RNA-seq libraries were prepared using 7-day-old plant roots, as described by [32]. Following the decapitation of additional roots, samples were collected after three hours, sixteen hours, and forty-six hours. To maintain consistency with expression variation depending on time and condition, we compared root samples from uncut roots and root tissue samples taken three hours after decapitation.

In addition to the above example, we included additional analyses that combine data from 88 microarray samples [52]. These samples were divided into three groups to compare the effects of different types of stress on gene expression. The first group was the control group (GSE5620). These samples were not exposed to any stress and were used to compare the other two groups. The second group (GSE5624) was exposed to drought stress, and the third group (GSE5623) was exposed to salt stress. The researchers wanted to see how drought and salt stress affect gene expression (Figures S1–S3) (Table S2).

Note that if this protocol is to be applied to species other than *A. thaliana* (e.g., other plants or animals), then the raw datasets must be carefully annotated to match the IDs across the co-expression and TF–target network.

The POTFUL protocol can be executed in six steps, which includes sample collection, high-throughput sequencing analysis, co-expression network, WGCNA modules, GRN inference, and functional enrichment analysis. These sections are discussed in more detail below.

The POTFUL GitHub repository can be cloned using the following command if using a Linux/Unix operating system, or the repository can be downloaded as a zipped folder from GitHub (see Appendix A).

$ git clone https://github.com/<user_id>/POTFUL.git

$ cd POTFUL

Use the following command to set up a conda virtual environment (‘POTFUL.yml’) and install all required packages. Additional .yml files are provided in the repository for Windows and Mac users, and in addition to Python packages, RUST must be installed.

$ conda env create -n POTFUL --file POTFUL.yml

The pipeline is divided into six major steps. Below is a step-by-step description of each step.

### 4.1. Choosing Plant Materials and Growing Conditions for High-Throughput Sequencing Analysis

**Duration**: Couple of weeks

Step 1 establishes the foundation for the anticipated outcome, i.e., the similarity or dissimilarity of the regulatory pattern between the two samples. It could take a couple of weeks from sowing seeds to collecting samples at the desired development stage, to preparing a library, to sequencing the results. Though in this study, a public dataset is used, GSE74488, uncut root samples (30 samples) were compared with root samples taken three hours after decapitation (67 samples). WGCNA requires a minimum of 15 samples, and 20 is the recommended sample number to be able to construct a co-expression network [53]. Even though the minimum number of samples for GENIE3 [38] is not explicitly recommended, it has been observed that recall increases as the number of samples increases [54].

### 4.2. High-Throughput Sequencing Analysis Data

**Duration**: 2 days

Depending upon the previous step, a high-throughput sequencing analysis is needed. As mentioned above, the expression values (GSE74488: GSE74488_sc_expression.csv.gz) were obtained from the Gene Expression Omnibus (GEO) repository for this study. Thus, no NGS analysis was required. For this framework to work, besides the NGS sequencing analysis, a proper annotation is paramount (see Appendix C, problem 1). For interspecies analysis, it is essential to perform the ortholog analysis once NGS analysis has been completed, and assign unique identifiers to each pair of orthologs of two species before proceeding to pairwise comparisons for inter-species samples.

### 4.3. Co-Expression Network and WGCNA Modules

**Duration**: 2 h

Following the standard WCGNA protocol, two co-expression networks were constructed based on the uncut and 3-h post-cut expression profiles. As the scale-free topology fit index for both the uncut and decapitated samples failed to reach values above 0.8 for reasonable powers, the soft-thresholding was set to 16 for the uncut and 12 for the 3 h decapitated samples [53]. The modules were identified using the WGCNA dynamic tree cut algorithm. To identify the modules, deepSplit and minClusterSize were the required parameters. Cluster splitting sensitivity was controlled by either a logical or integer (0–4) value for deepSplit [37]. A standalone module must have a minimum number of genes, which is controlled by minClusterSize. For both datasets, the deepSplit parameter was set to zero, whereas minClusterSize was set to 300 and 250 for the uncut and 3 h decapitated samples, respectively. The edges and node table need to be exported as the tab-separated text file format to be able to load in POTFUL using the exportNetworkToCytoscape function, and the adjacency threshold for including edges in the output was set to 0.9.

### 4.4. Inference of Gene Regulatory Network

**Duration**: 15 min

The expression profile and grnboost2 [38] were used to infer the weighted gene regulatory network. In this case study, we used the Python implementation of grnboost2 for inferring the GRN using the TF list and expression data (https://arboreto.readthedocs.io/en/latest/index.html (accessed on 29 April 2023)). The GRN can be loaded as a tab-separated text file in POTFUL. Below is an example script regarding GRN analysis using grnboost2.
#!/usr/bin/env python3 import pandas as pd from arboreto.algo import grnboost2, genie3 from arboreto.utils import load_tf_names from distributed import LocalCluster, Client tfdf = pd.read_csv(“Auxiliary_File/Arabidopsis_TF and family.csv”) tf_names = list(set(tfdf[‘Protein ID’].values.tolist())) len(tf_names) ex_matrix = pd.read_csv(“1_Expression_data/Expr_Uncut.csv”, sep=‘,’, index_col=0).T ex_matrix.head() local_cluster = LocalCluster(n_workers=10,                                                        threads_per_worker=1,                                                        memory_limit=8e9) custom_client = Client(local_cluster) network = grnboost2(expression_data=ex_matrix,                                   tf_names=tf_names, verbose=True, client_or_address=custom_client) network.to_csv(‘3_GRN_data/GSE74488_Uncut_arboreto_regnet.tsv’, sep=‘\t’, index=False) network.head()

**Note**: The auxiliary files are provided in the GitHub repository, and before moving to the next step, cloning the repository was performed as described earlier.

### 4.5. WGCNA Module Enrichment

**Duration**: 5 min

Below is an example of a module-to-module comparison of the WGCNA module of the uncut samples with the 3 h decapitated root samples. After the successful execution of the previous steps, three resulting files were expected (WGCNA node table, WGCNA edge table, and GRN data) for each dataset. All of the files were verified to be in the correct format and in the current directory or path.

Note that as per convenience, using an appropriate integrated development environment (IDE) is recommended, such as Jupyter notebook, JupyterLab, or Visual Studio Code, in order to execute all of the following Python scripts.

a. The following command was used to activate the conda environment:
  $ conda activate POTFUL

b. The POTFUL (v v1.0.1) package was loaded:
from POTFUL import POTFUL POT = POTFUL()

c. All of the auxiliary files were loaded using the following command:
POT.Load_Auxiliary_Files(WGCNA_COLOR_MAP=“Auxiliary_File/WGCNA_COLOR_MAP.csv”,                                              TF_Targets=“Auxiliary_File/masterTF-target.txt”,                                               TF_Family=“Auxiliary_File/Arabidopsis_TF and family.csv”)

d. The pre-analyzed (WGCNA and GRN files) files for both datasets were loaded, the uncut, and 3hpc, using the following command:
# Uncut POT.Load_Files(Sample_name=“Uncut”,                         NODE_File=“2_WGCNA_data/WGCNA_GSE74488_Uncut/Nodes_Uncut.txt”,                         EDGE_File=“2_WGCNA_data/WGCNA_GSE74488_Uncut/Edges_Uncut.txt”,                         GRN_File=“3_GRN_data/GSE74488_Uncut_arboreto_regnet.tsv”) # 3hr decapitated root samples POT.Load_Files(Sample_name=“3hpc”,                         NODE_File=“2_WGCNA_data/WGCNA_GSE74488_3hpc/Nodes_3hpc.txt”,                         EDGE_File=“2_WGCNA_data/WGCNA_GSE74488_3hpc/Edges_3hpc.txt”,                         GRN_File=“3_GRN_data/GSE74488_3hpc_arboreto_regnet.tsv”) # Uncut

**Critical**: The datasets were verified to have loaded correctly, and their index numbers were printed for future reference (i.e., 0: Uncut and 1: 3hpc) using the following Python script:
Samples = POT.Samples for i in range(len(Samples)):        print(i, Samples[i]) *0 Uncut* *1 3hpc*

e. As part of the enrichment analysis, POTFUL uses Enrichr API (GSEApy); to be able to do so using WIGCNA modules, a GMT (Gene Matrix Transposed file format (*.gmt)) file was created. In the WGCNA module *.gmt file, each row consists of three components, first the name of the WGCNA module (e.g., turquoise, tan, etc.), then the description (e.g., WGCNA3hpc, WGCNAunct, etc.), and finally the list of all of the genes in the module. A *.gmt file was created for both samples for enrichment analysis using the following function for each dataset:
POT.WGCNA_Bucket_GMT() GMT_base/POTFUL-Uncut.gmt 8921 GMT_base/POTFUL-3hpc.gmt 4756

**Note**: We verified that the GMT files were created and ready to load using the following command:
for i in range(len(Samples)):        print((POT.File[Samples[i]][‘GMT’])) *# GMT_base/POTFUL-Uncut.gmt* *# GMT_base/POTFUL-3hpc.gmt*

f. Using the following Python script, a bar chart of the numbers of the genes in each WGCNA module for each dataset was created (Figure 1a,b):
fig = POT.Plots[Samples[0]][‘WGCNA_BarPlot’] fig.show() *# Figure 1a* fig = POT.Plots[Samples[1]][‘WGCNA_BarPlot’] fig.show() # Figure 1b

**Note**: The “fig” is a Plotly figure object that can be further modified accordingly to export a publication quality image, as described below:
fig.update_layout(autosize=False, width=350, height=400,         xaxis_title=“WGCNA Module”, yaxis_title=“Number of genes”,         plot_bgcolor = ‘rgba(0, 0, 0, 0)’,         font=dict(family=“Times New Roman”, size=10, color=“black”)) fig.update_xaxes(showline=True, linewidth=2, linecolor=‘black’, mirror=True) fig.update_yaxes(showline=True, linewidth=2, linecolor=‘black’, mirror=True) fig.write_image(“POTFUL_OUT/Uncut.png”, scale=2) fig.write_image(“POTFUL_OUT/Uncut.svg”, scale=2)

g. Using Fisher’s exact test, the *p*-value was calculated (hypergeometric test), indicating whether the overlap between the two module gene lists is significant. As the background parameter, the nodes of both co-expression networks that were being compared were used. For assigning significance color codes and significance asterisks, only ‘Adjusted *p*-value’ is considered by default. An enrichment analysis of modules was performed of one sample concerning another sample using the following command:
POT.WGCNA_Module_Enrichment(Samples[0], Samples[1])

**Note**: The results of the module enrichment analysis can be accessed as a Python (Pandas) dataframe using the following command:

print(POT.Data[“Enrichment_Dotplot”])

h. Using the following Python command, the enrichment dot plot was generated, and a high-quality image was exported. Every dot in the enrichment dot represents the significance of the enrichment, i.e., green (***), gold (**), and yellow (*). In contrast, the plus (+) symbol represents not significantly enhanced sets.
fig = POT.Plots[“Enrichment_Dotplot”] fig.update_layout(         autosize=False,         width=490,         height=500, font=dict(         family=“Arial”,         size=12,         color=“black”)) fig.write_image(POT.OutDir+f”3hpc__UncutEnri_dot.png”, scale=2) fig.write_image(POT.OutDir+f”3hpc__UncutEnri_dot.svg”) *# Figure 1c*

**Note**: Each WGCNA module of samples on the y-axis (uncut) was compared to samples on the x-axis (3hpc). The order of samples in the “WGCNA_Module_Enrichment” function was changed to do the comparison in the other direction, i.e., (uncut vs. 3hpc) (i.e., POT.WGCNA_Module_Enrichment(Samples[1], Samples [0])). Additionally, the “WGCNA_Module_Enrichment” function only accepts two samples.

### 4.6. Co-Expression and GRN Sample Overlap

**Duration**: 5 min

a. The TF–target pairs that did not belong to the known curated TF–target pairs were filtered out using the following Python command for each sample:
# Uncut POT.TF_reg(Samples[0], Filter=1) # 3hpc POT.TF_reg(Samples[1])

**Note:** We could choose whether to do this step or not. We included this choice to help deal with large numbers of TF–target pairs created by prediction tools like GENI3. The purpose of removing some pairs is to make the analysis smoother, especially when there are many of pairs to go through.

b. Using the following Python command, the remaining GRN-weighted network was matched with the co-expression network to keep only those pairs that are co-expressed and involved in regulation:
# Uncut POT.merge_reg_coexp(Samples[0]) # 3hpc POT.merge_reg_coexp(Samples[1])

**Note**: The network of node pairs that are co-expressed and are TF–target pairs is called the co-expressed–GRNs.

c. Network centrality analysis was performed on the co-expressed–GRN using the following command (see Appendix C, Problem 4):
# Uncut POT.network_centrality(Samples[0]) # 3hpc POT.network_centrality(Samples[1])

**Note**: Although this step was optional, it is recommended.

d. The GraphML file was generated, and the network visualized using the following command (see Appendix C, Problem 3):
# Uncut POT.generate_graphml_out(Samples[0]) # 3hpc POT.generate_graphml_out(Samples[1])

e. The CERN was plotted and exported using the following command:
# Uncut POT.Graph_vis(Samples[0]) POT.Plots[Samples[0]][‘Network_Viz’] .show(POT.OutDir+’Uncut.html’) *# Figure 1d* # 3hpc POT.Graph_vis(Samples[1]) POT.Plots[Samples[1]][‘Network_Viz’] .show(POT.OutDir+’3hpc.html’) *# Figure 1e*

f. The co-expressed–GRNs of both samples were compared and plotted to check for any overlapping nodes, using the following command:
POT.netowork_overlap(Samples[0], Samples[1]) *# There are 20 nodes overlapping between pair of Graphs* *(‘AT5G41920’, ‘AT1G58340’, ‘AT1G18330’, ‘AT2G42150’, ‘AT3G03200’, ‘AT3G04030’, ‘AT1G51220’, ‘AT5G62320’, ‘AT2G45660’, ‘AT1G75390’, ‘AT5G42070’, ‘AT4G08940’, ‘AT3G10113’, ‘AT3G01530’, ‘AT1G75820’, ‘AT1G75388’, ‘AT2G18380’, ‘AT4G36900’, ‘AT5G46590’, ‘AT2G45420’)* POT.Plots[‘Overlap_Network_Viz’].show(‘Overlap.html’) *# Figure 1f*

## Data Availability

The POTFUL package as well as examples of the output can be found on GitHub (https://github.com/nilesh-iiita/POTFUL (13 February 2023), https://nilesh-iiita.github.io/POTFUL/3_POTFUL_Example.html (13 February 2023)) and Zenodo (https://doi.org/10.5281/zenodo.7901425 (13 February 2023)).

## Supplementary Materials

The following supporting information can be downloaded at: https://www.mdpi.com/article/10.3390/plants12203618/s1, Table S1: Co-expression–GRN uncut and 3 h post-decapitation overlap. Table S2: Co-expression–GRN for control, salt, and drought. Figure S1: WGCNA modules of co-expression network and module to module comparisons. Bar plot displaying the number of nodes in each WGCNA co-expression network module (a–f). Comparative module-to-module enrichment analysis of drought and salt co-expression networks in shoot (g) and root (h) samples. Significant and high-priority enriched pairs are highlighted with red dotted squircles. In the enrichment dot-plot, three colors of dots/circles represent the significance levels of enrichment: green for *p* < 0.001, gold for *p* < 0.01, and yellow for *p* < 0.05; Figure S2: WGCNA module-to-module control vs shoot and root comparison. Comparative module-to-module enrichment analysis of drought and salt co-expression networks in shoot (g) and root (h) samples with the control co-expression network (a–d). Significant and high-priority enriched pairs are highlighted with red dotted squircles. Within the enrichment dot-plot, the significance levels of enrichment are indicated using three distinct colors of dots/circles: green for *p* < 0.001, gold for *p* < 0.01, and yellow for *p* < 0.05; Figure S3: Co-Expressed-Regulatory Network (CERN) comparison. Network overlap of CERNs with respect to control, drought, and salt sample types. Exclusive nodes are shown in either green or gold, while common nodes are shown in yellow. The network is plotted using the Gephi tool. References [30,52] are cited in the supplementary materials.

## Appendix A

POTFUL analysis is explained in a comprehensive set of online tutorials (https://nilesh-iiita.github.io/POTFUL (13 February 2023), https://nilesh-iiita.github.io/POTFUL/3_POTFUL_Example.html (13 February 2023)). The tutorials provide Python script in Jupyter notebook format along with comments and explanations of both the input and output. Using a Jupyter notebook or an IDE such as Microsoft Visual Studio Code, the user can execute the code.

## Appendix B

**Limitations**

- The protocol enables the comparison of pairs of co-expression networks in a flexible manner.
- TF–target networks are necessary for the construction of co-expressed–GRNs. POTFUL may not be applicable for non-model plants without a robust list of TFs.
- Different cell types regulate genes differently, and the pattern changes over time. The protocol cannot be applied to samples of different types of cells.

## Appendix C

**Troubleshooting**

**Problem** 1: Microarray probe annotation

Potential Solution

The annotation of probesets is very important before inferring the GRN, since it would be impossible to map co-expressed genes with TF–targets if the same ID system were used. In general, annotation information is provided in the microarray data softfile (GEO). The annotation of DNA microarray experiments can be carried out with annotation tools, an R package, or similar tools.

**Problem** 2: Number of co-expression modules/clusters

Potential Solutions

For a balanced comparison, both co-expression networks should have equal or almost equal numbers of WGCNA modules. To adjust the number of WGCNA modules, use the minClusterSize parameter in the cutreeDynamic R function described in the WGCNA tutorial.

**Problem** 3: Visualization of Network/Graph

Potential Solutions

GraphML files generated by this protocol can be imported into external tools such as Gephi and Cytoscape for visualization.

**Problem** 4: Network centrality analysis

Potential Solutions

The network centrality analysis can be performed using the POT.network_centrality() function. By default, only three centrality analyses are set to be performed. For additionally required centrality analyses, apply the networkx or custom function on the network, i.e., POT.Data[<“Sample_name”>][<“Network”>].

## Appendix D

**Performance**

POTFUL is a protocol designed to integrate the co-expression and transcription factor target protein networks to infer gene regulation in plants. It is implemented in Python 3, which is a popular programming language used in scientific research. The protocol is designed to be reproducible, meaning that it can be used by other researchers to perform similar analyses and obtain comparable results. One potential advantage of POTFUL is that it allows researchers to integrate multiple types of data, including transcriptome datasets and protein–protein interaction networks, in order to gain insights into gene regulation. The protocol also incorporates statistical methods to identify significant co-expression relationships and transcription factor target protein interactions, which can help researchers prioritize genes and pathways for further investigation.

The POTFUL protocol can be divided into multiple steps, which includes sample collection, high-throughput sequencing analysis, co-expression network, WGCNA modules, GRN inference, functional enrichment analysis, and visualization. The performance constraints of POTFUL depend on several factors, such as the size of the input data, the computational resources available, and the efficiency of the algorithms used. For example, the POTFUL protocol requires the analysis of high-throughput sequencing data, which can be computationally intensive, especially for larger datasets. The pipeline also relies on several algorithms for network inference and analysis, such as grnboost2 and WGCNA, which can have different computational requirements depending on the size of the input data. In addition, the performance of POTFUL can be affected by the computational resources available, such as the processing power of the computer or the availability of high-performance computing (HPC) clusters. The protocol has been tested on a range of devices, including computers with Intel i7 processors and 16 GB of RAM, but it may require more powerful hardware for larger datasets or more complex analyses. Finally, the efficiency of the algorithms used in POTFUL can also affect its performance. While the protocol relies on established algorithms and software packages, some steps may require additional tuning or optimization for specific datasets or research questions.
