# Supplementary material for: Integrated Systems Biology Pipeline to Compare Co-Expression Networks in Plants and Elucidate Differential Regulators
Source: Plants (Basel). 2023 Oct 19;12(20):3618. doi: 10.3390/plants12203618 (PMC10610404; doi:10.3390/plants12203618)
Supplement: Supplementary file 1 [file plants-12-03618-s001.zip › supplementary material.pdf]

Supplementary text

A total of 88 plant samples [1] were analyzed using the WGCNA [2] package to infer the co-expression network and to investigate the effects of different stress conditions on the plant's growth and development. The samples were divided into three distinct groups: a control group (GSE5620), a drought (GSE5624) stress group, and a salt stress group (GSE5623).

The control group, consisting of 36 samples, served as the baseline for comparison. Within this group, there were 18 root samples and 18 shoot samples, carefully selected to represent a healthy and normal growth state of the plants under optimal conditions. These samples provided crucial data for understanding the typical growth patterns of the plants.

For the drought stress group, 28 samples were subjected to a controlled drought stress environment. This simulated condition aimed to mimic the adverse effects of limited water availability on plant growth. Among these samples, 14 were root samples and 14 were shoot samples. The data collected from these stressed samples allowed for a comprehensive analysis of how plants respond and adapt to drought stress at both root and shoot levels.

Similarly, the salt stress group was comprised of 24 samples. These samples were exposed to controlled salt stress conditions to simulate the challenges posed by elevated salt levels in the soil. Within this group, there were 14 root samples and 14 shoot samples. Analyzing the responses of the plants under salt stress provided valuable insights into how plants cope with saline environments and the subsequent impact on root and shoot growth.

Using the WGCNA [2] package 6 co-expression networks were inferred, control-root (5,505 nodes and 25,497 edges), control-shoot (10,972 nodes and 172,990 edges), drought-root (1,975 nodes and 4,256 edges), drought-shoot (8,111 nodes and 107,980 edges), salt-root (11,816 nodes and 98556 edges) and salt-shoot (21,591 nodes and 2,916,475 edges). The co-expressions network was compared module-to-module using the POTFUL tool tissues wise, control co-expression network was compared with drought and salt co-expression networks of same tissue type that is root with root and shoot with shoot (Fig.S2). Further, the drought Co-expression of network were also compared with co-expression network of salt samples (Fig.S1).

The Co-Expressed-Regulatory Network (CERN) comparison analysis revels that there is significant overlap between drought and control regardless of tissue type (shoot and root) Fig.S3. Whereas salt network doesn’t overlap significancy with control or with drought network in case of root sample as compared to shoot networks. Gephi 0.10.1 was used to visualize the network to display flexibility of POTFUL (Table S2).

Supplementary figure

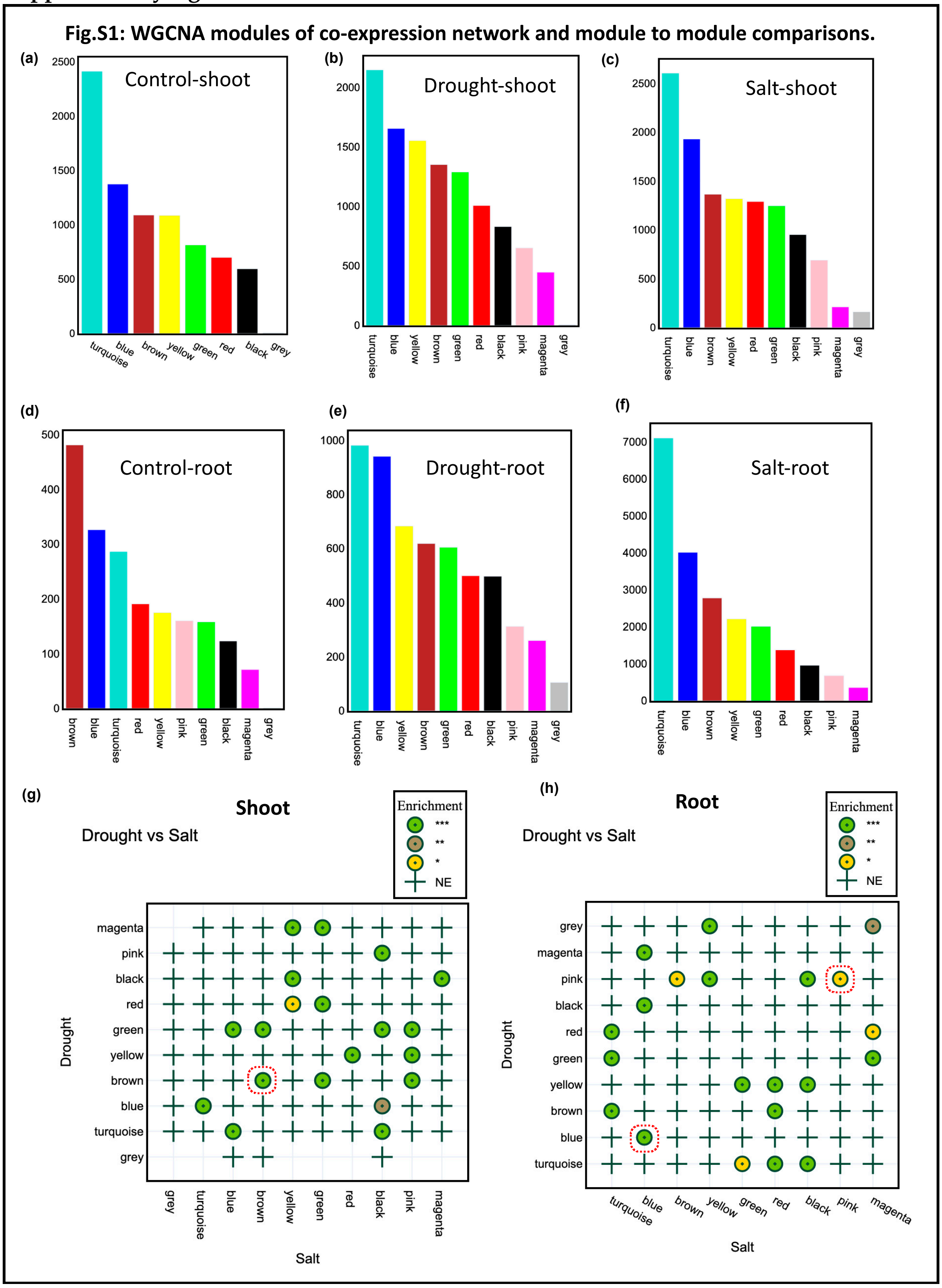

Figure S1: WGCNA modules of co-expression network and module to module comparisons. Bar plot displaying the number of nodes in each WGCNA co-expression network module (a-f). Comparative module-to-module enrichment analysis of drought and salt co-expression networks in shoot (g) and root (h) samples. Significant and high-priority enriched pairs are highlighted with red dotted squircles. In the enrichment dot-plot, three colors of dots/circles represent the significance levels of enrichment: green for $p<0.001$, gold for $p<0.01$, and yellow for $p<0.05$.

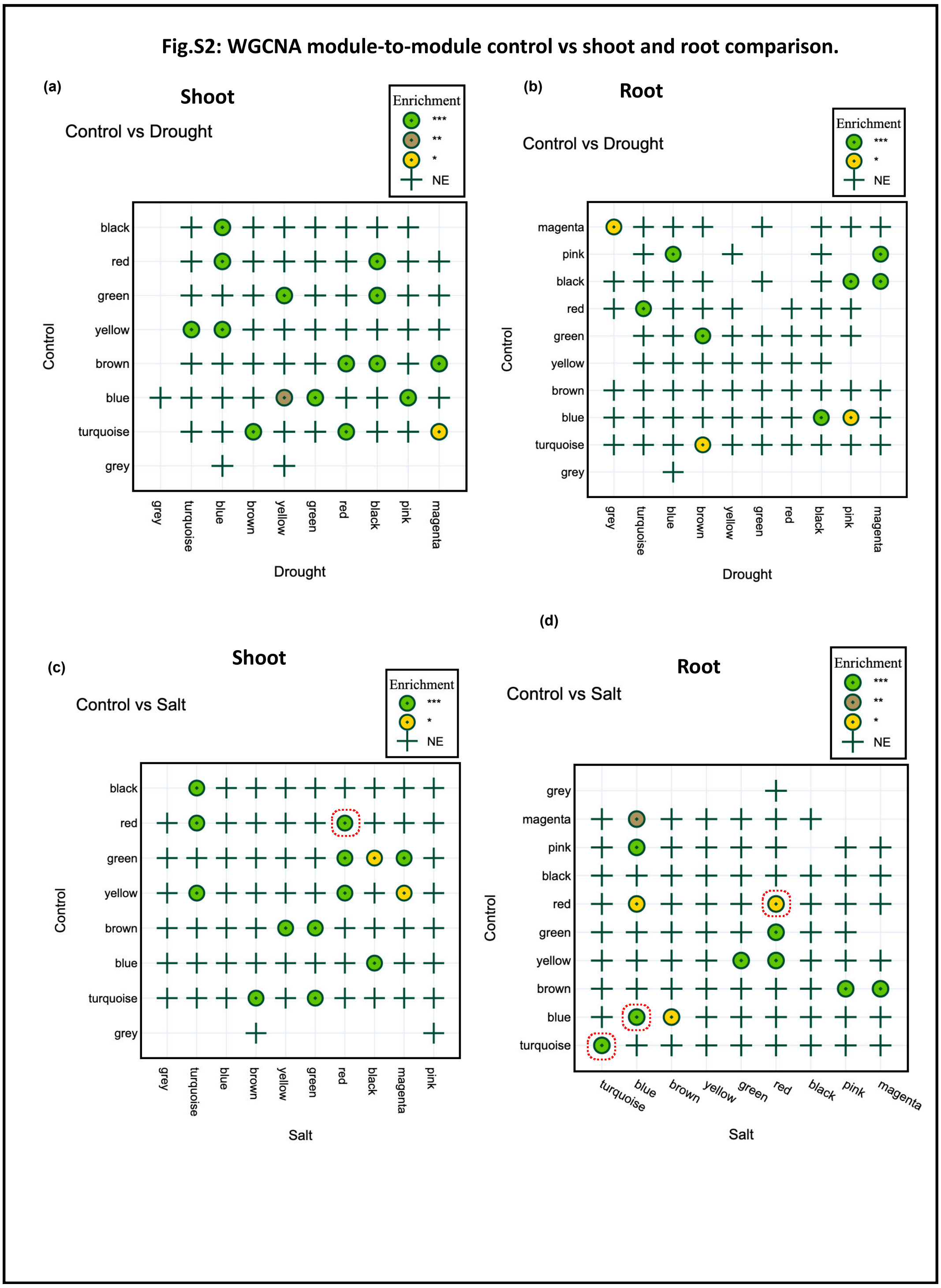

Figure S2: WGCNA module-to-module control vs shoot and root comparison. Comparative module-to-module enrichment analysis of drought and salt co-expression networks in shoot (g) and root (h) samples with the control co-expression network (a-d). Significant and high-priority enriched pairs are highlighted with red dotted squircles. Within the enrichment dot-plot, the significance levels of enrichment are indicated using three distinct colors of dots/circles: green for p<0.001, gold for p<0.01, and yellow for p<0.05.

Fig.S3: Co-Expressed-Regulatory Network (CERN) comparison.

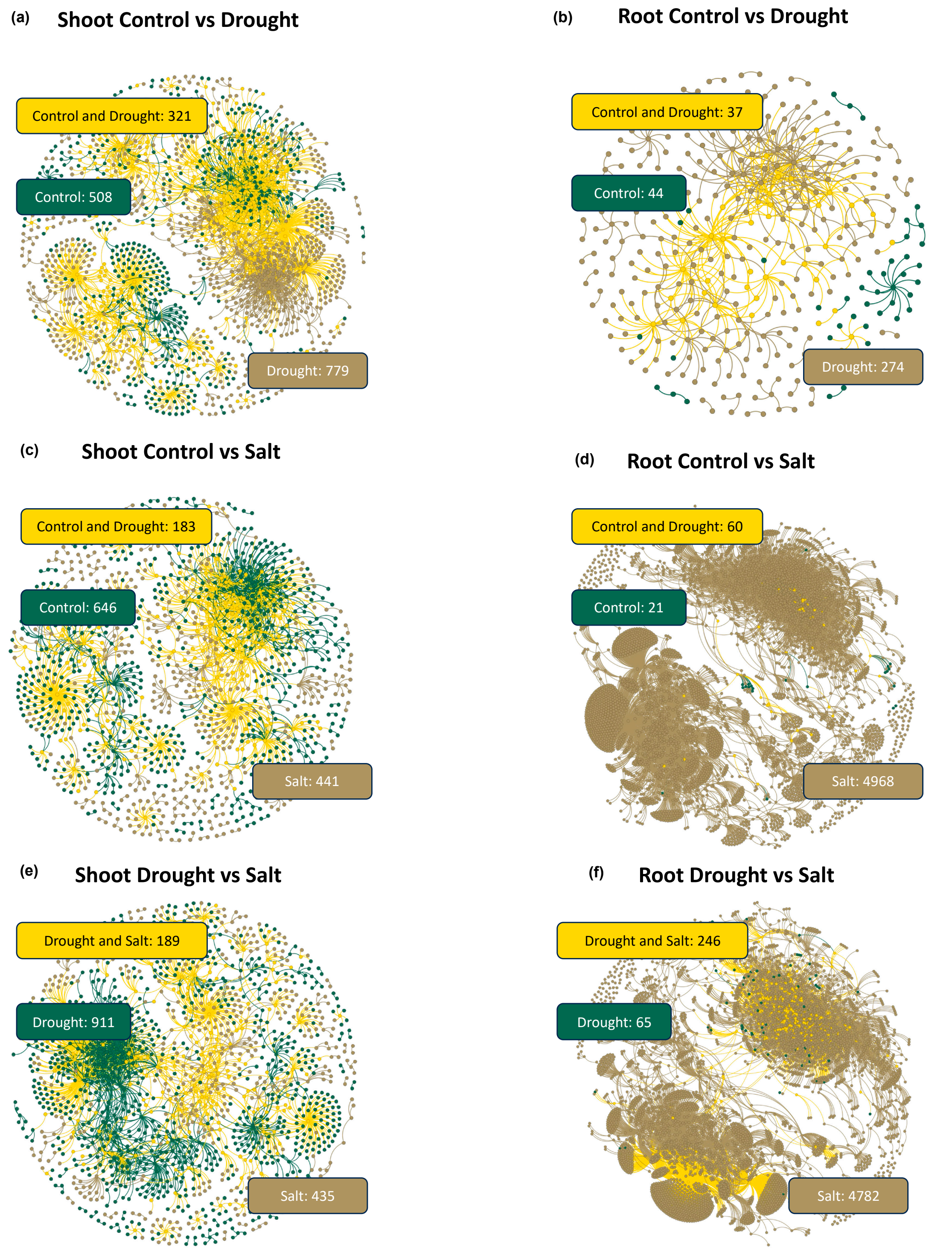

Figure S3: Co-Expressed-Regulatory Network (CERN) comparison. Network overlap of CERNs with respect to control, drought, and salt sample types. Exclusive nodes are shown in either green or gold, while common nodes are shown in yellow. The network is plotted using the Gephi tool.

1. Kilian, J.; Whitehead, D.; Horak, J.; Wanke, D.; Weinl, S.; Batistic, O.; D'Angelo, C.; Bornberg-Bauer, E.; Kudla, J.; Harter, K. The AtGenExpress global stress expression data set: protocols, evaluation and model data analysis of UV-B light, drought and cold stress responses. *Plant J* **2007**, *50*, 347-363, doi:10.1111/j.1365-313X.2007.03052.x.
2. Langfelder, P.; Horvath, S. WGCNA: an R package for weighted correlation network analysis. *BMC Bioinformatics* **2008**, *9*, 559, doi:10.1186/1471-2105-9-559.
